# Identification of stage-specific genes associated with lupus nephritis and response to remission induction in NZB/W and NZM2410 mice

**DOI:** 10.1186/ar4637

**Published:** 2014-09-18

**Authors:** Ramalingam Bethunaickan, Celine C Berthier, Weijia Zhang, Ridvan Eksi, Hong-Dong Li, Yuanfang Guan, Matthias Kretzler, Anne Davidson

**Affiliations:** 1 Center for Autoimmunity and Musculoskeletal Diseases, Feinstein Institute for Medical Research, Manhasset, NY, USA; 2 University of Michigan, Ann Arbor, MI, USA; 3 Mount Sinai Medical Center, New York, NY, USA

## Background

Lupus nephritis affects 30 to 70% of systemic lupus erythematosus (SLE) patients and its treatment remains insufficiently effective and excessively toxic. Although biomarkers for nephritis are being identified there is still no reliable way of predicting an impending renal flare or determining which patients will respond to therapy. Because human renal tissue cannot be obtained sequentially during remission and relapse, animal models are often used to study progression of lupus nephritis. To elucidate the molecular mechanisms involved in renal inflammation during the progression, remission and relapse of nephritis we performed a transcriptome analysis of renal tissue from two murine lupus models, NZB/WF1 mice that develop proliferative glomerulonephritis and NZM2410 mice that develop glomerulosclerosis with minimal inflammation.

## Methods

Kidneys from NZB/W F1 and NZM2410 mice were harvested at intervals during their disease course or after remission induction with either combination cyclophosphamide/costimulatory blockade or with BAFF inhibition. Genome-wide expression profiles were obtained from microarray analysis of perfused kidneys. Real-time PCR analysis for selected genes was used to validate the microarray data. Comparisons between groups using SAM, and unbiased analysis of the entire dataset using singular value decomposition and self-organizing map were performed.

## Results

Few changes in the renal molecular profile were detected in pre-nephritic kidneys but a significant shift in gene expression, reflecting inflammatory cell infiltration and complement activation, occurred at proteinuria onset. Subsequent changes in gene expression predominantly affected mitochondrial dysfunction and metabolic stress pathways. Remission induction reversed most, but not all, of the inflammatory changes and progression towards relapse was associated with recurrence of inflammation, mitochondrial dysfunction and metabolic stress signatures. Endothelial cell activation, tissue remodeling and tubular damage were the major pathways associated with loss of renal function.

## Conclusions

Immune cell infiltration and activation is associated with proteinuria onset and reverses with immunosuppressive therapy but disease progression is associated with renal hypoxia and metabolic stress. Optimal therapy of SLE nephritis may therefore need to target both immune and nonimmune disease mechanisms. In addition, the overlap of a substantial subset of molecular markers with those expressed in human lupus kidneys suggests potential new biomarkers and therapeutic targets.

